# ABCC6 Knockdown Fuels Cell Proliferation by Regulating PPARα in Hepatocellular Carcinoma

**DOI:** 10.3389/fonc.2022.840287

**Published:** 2022-02-24

**Authors:** Zhicong Zhao, Zhenjun Zhao, Jianye Wang, He Zhang, Zhifeng Xi, Qiang Xia

**Affiliations:** ^1^ Department of Liver Surgery, Renji Hospital, Shanghai Jiao Tong University School of Medicine, Shanghai, China; ^2^ Department of Systems Biology, Beckman Research Institute of City of Hope, Duarte, CA, United States; ^3^ Department of Surgery, School of Medicine, Technical University of Munich, Munich, Germany; ^4^ Department of Surgery, The University of Hong Kong-Shenzhen Hospital, Shenzhen, China; ^5^ Shanghai Engineering Research Center of Transplantation and Immunology, Shanghai, China; ^6^ Shanghai Institute of Transplantation, Shanghai, China

**Keywords:** ABC transporter family, ABCC6, biomarker, cell proliferation, hepatocellular carcinoma, PPARα, peroxisome

## Abstract

The ATP binding cassette (ABC) transporter family is ubiquitous in eukaryotes, specifically in vertebrates, and plays a crucial role in energy homeostasis, cell signaling, and drug resistance. Accumulating evidence indicates that some ABC transporters contribute to cancer cell proliferation and tumor progression; however, relatively little is known about the behavior of the ABC transporter family in hepatocellular carcinoma (HCC). By analyzing two public transcriptomic databases, we evaluated the effect of genes in the ABC transporter family on HCC prognostic prediction; ABCC6 was selected for further study. Notably, ABCC6 was found to be downregulated in HCC tissues and correlated with favorable outcomes in patients with HCC. Moreover, *ABCC6* knockdown not only significantly promoted cell proliferation *in vitro* and *in vivo*, but also inhibited cell cycle arrest and cell apoptosis. Transcriptome analysis revealed that *ABCC6* depletion enhanced the “mitotic cell cycle” and “DNA replication” pathways, and suppressed the “PPAR signaling pathway”. Further investigation demonstrated that PPARα, one of the key regulators in peroxisome metabolism, is located downstream of ABCC6. In summary, our study provides profound insights into the behavior of ABC transporter family genes in various HCC cohorts, identifies ABCC6 as a biomarker for early-stage HCC diagnosis, and offers experimental basis for further investigations of targeting ABCC6 in the treatment of patients with HCC.

## Introduction

Hepatocellular carcinoma (HCC) is the sixth most common cancer worldwide and accounts for approximately 85% of primary liver cancers (1). Moreover, incidence rates of HCC are significantly higher in China, Southeast Asia, and Africa than the rest of the world (2). Risk factors for HCC include hepatitis B virus infection, cirrhosis, non-alcoholic fatty liver disease, excessive alcohol consumption, autoimmune disease, and accumulation of reactive oxygen species (ROS) (3, 4). Additionally, early diagnosis through surveillance and curative treatment has considerably improved the 5-year survival of patients with HCC (5). Therefore, it’s strongly recommended to systematically screen target populations that are at a particularly high risk for developing HCC.

The ATP binding cassette (ABC) transporter family represents one of the largest families of transmembrane proteins (6). ABC transporters are found among all living organisms, and are classified into seven subfamilies, designated ABCA-ABCG (6). By utilizing the energy from ATP hydrolysis, ABC transporters can translocate specific substrates across the membrane (7). Furthermore, the past few decades have witnessed numerous studies documenting the contribution of ABC transporters to multidrug resistance. Specifically, some ABC transporters, such as ABCC1, ABCG2, and ABCB1, are highly efficient at extruding drugs from cancer cells, resulting in substantial multidrug resistance (8). More recently, accumulating evidence indicates that some ABC transporters also contribute to cancer cell proliferation and tumor progression (6, 7). For example, *ABCC4* knockdown induces cell cycle arrest and apoptosis in acute myeloid leukemia (9). Overexpression of *ABCC1* correlates with reduced overall survival in patients with neuroblastoma (10). Elevated *ABCA13* mRNA levels are linked to poor clinical outcomes in patients with gastric adenocarcinoma (11). At the same time, some ABC transporters (e.g., ABCA1, ABCA2, and ABCA7) are essential for lipid transport and homeostasis (12). However, because ABC transporters perform multiple functions simultaneously, controversial results have been reported (13). Thus, there is a pressing need to understand the overall behavior of ABC transporter family genes in HCC.

ABCC6, primarily localized in the basolateral membrane of hepatocytes, belongs to the ABC transporter C subfamily (14). Several studies have demonstrated that ABCC6 plays an important role in mineralization homeostasis. It’s well-established that ABCC6 mutations cause a complex autosomal recessive disease, called pseudoxanthoma elasticum (PXE) (15, 16). Mechanistically, ABCC6 overexpression results in the efflux of ATP, which is rapidly converted into nucleoside monophosphates and pyrophosphate and alters the extracellular environment (17). Moreover, recent studies have suggested that ABCC6 variations correlate with altered plasma triglyceride levels and increased coronary risks (18, 19). Additionally, genetic deletion of *Abcc6* in mice disturbs cholesterol homeostasis and lipid metabolism (20). However, the role of ABCC6 in tumor biology has been poorly investigated.

In the present study, we first analyzed the prognostic value of ABC transporter family genes using the Cancer Genome Atlas (TCGA) and GSE14520 datasets, and then identified ABCC6 as a potential biomarker for early-stage HCC diagnosis and prognostic prediction. Specifically, ABCC6 expression was downregulated in HCC tissues and positively correlated with favorable overall survival in patients with HCC. Functional studies further revealed that ABCC6 knockdown significantly enhanced cancer cell proliferation *in vitro* and *in vivo*. Mechanistically, ABCC6 depletion inhibits the PPARα (Peroxisome proliferator-activated receptor alpha) activity and protects HCC cells from oxidative damage.

## Materials and Methods

### Cell Culture

HCC Cell lines MHCC97H, SMMC7721, and Huh7 were obtained from Cell Resource Center of Shanghai Institutes for Biological Sciences, Chinese Academy of Sciences. Normal human hepatic cell line L-02 and human HCC cell lines MHCC97L and LM3 were kindly given from Huashan Hospital, Cancer Metastasis Institute, Fudan University, Shanghai. All cell lines were grown in DMEM medium (Gibco) supplemented with 10% Fetal bovine serum(FBS)(Gibco), and 1% penicillin-streptomycin (Gibco), incubated at 37°C and 5% CO_2_. Mycoplasma contamination was tested every month.

### Patients and Samples

Fresh human HCC tumor tissues and adjacent paratumor tissues were collected from patients who underwent surgical resection or liver transplantation at Department of Liver Surgery, Renji Hospital, Shanghai Jiao Tong University School of Medicine. Written informed consent was obtained from each patient. For Clinical analyses, two independent HCC cohorts of HCC patients were retrospectively adopted. HCC cohort 1 (hepatectomy cohort) included 153 HCCs collected between February 2010—August 2015 from Department of Liver Surgery, Renji Hospital, Shanghai Jiao Tong University School of Medicine. HCC cohort 2 (liver transplantation cohort) included 70 HCCs collected between January 2015—April 2016 from Department of Liver Surgery, Renji Hospital, Shanghai Jiao Tong University School of Medicine. All patients were diagnosed with HCC according to the NCCN guidelines and routinely followed up. This study was approved by the Renji Hospital Ethics Committee.

### Constructs and Transfections

Human full-length *ABCC6* cDNA was inserted to the lentiviral vector CMV-MCS-3FLAG-SV40- EGFP-IRES-Puromycin plasmid by Fubio company (Shanghai, China). Short hairpin RNA (shRNA) targeting ABCC6 was constructed into PLKO.1 plasmid by Genepharma Company (Shanghai, China), while a non-target shRNA (5’- GCGCGCTTTGTAGGATTCG-3’) was used as a negative control. To construct stable cell lines with overexpression or downregulation of *ABCC6*, lentivirus particles were generated based on above plasmids and transfected into HCC cell lines in the presence of polybrene. 48 hours after infection, puromycin (2μg/ml) was added into culture cells. Knockdown and overexpression efficiency was confirmed by western blot. The shRNA target sequences employed in this study are listed as below: shABCC6#1: GTGGCCGAGAATGCTATGAAT; shABCC6#2: CGTAGATGAAAGCCAGAGGAT.

### Tissue Microarray Immunohistochemistry Analysis

Two tissue microarrays with definite HCC diagnosis were constructed in this study. All HCC samples were first reviewer histologically by hematoxylin and eosin staining to identify the border between Tumor and paratumor. Then, representative areas were punched out and mounted onto a recipient block with a semi-automated TMArrayer. Immunohistochemistry assays using tissue microarray were performed according to standard protocol. Briefly, paraffin-embedded tissue microarray slides were deparaffinized and rehydrated for 30 min. Antigen retrieval was done by incubating the slides in citrate buffer (pH 6.0) by boiling for 10 min in the microwave oven. After fully cooled down, the slides were incubated with ABCC6 antibody (Proteintech, 27848-1-AP) overnight at 4°C. After washing three times with PBST, the slides were incubated with HRP-conjugated secondary antibody (Proteintech, SA00001-2). Signals were developed in DAB detection solution (Beyotime, p0203) under microscopic observation and counterstained with hematoxylin. After staining, photographs were captured using Leica microscope. The Image-Pro Plus v6.0 software was used to calculate the integrated optical density (IOD) of each photograph, and the ratio of IOD to total tissue area was calculated as staining intensity.

### CCK-8, Colony Formation, and EdU (5-Ethynyl-2’-Deoxyuridine) Incorporation Assays

For CCK-8 assay, HCC cell lines were infected with indicated lentivirus and selected for 1 week to generate stable knockdown or overexpression cells. Then, the cells were seeded into 96-well plates at a density of 3 × 10^3^ cells per well and incubated at 37°C. Every 24 hours, 10% (V/V) CCK-8 (Dojindo, CK04-11) was added to the culture medium and incubated for one hour. Cell viability was measured at OD 450 nm using a SpectraMax i3 microplage reader (Molecular Devices, USA).

Colony formation assay were performed to evaluate the long-term proliferation ability. HCC cell lines were seeded into 60mm dishes at a density of 3 × 10^3^ cells per well and cultured for 14 days. The medium was changed twice a week. Cells were washed gently with PBS and fixed in 4% formaldehyde for 20 minutes followed by staining with 0.1% (w/v) crystal violet. The cell culture plates were scanned to obtain digital images, and the colonies were counted under microscope.

The EdU (5-Ethynyl-2’-Deoxyuridine) incorporation assay was performed using BeyoClick™ EdU Cell Proliferation Kit (Beyotime, C0078S) according to the manufacturer’s instructions to further validate cell proliferation ability. Briefly, 1×10^5^ cells were planted in 24-well plates with coverslips 24 hours before experiments. Then, 50 mM EdU labeling medium was added into each well and incubated for 2h at 37°C. The cells were fixed using 4% paraformaldehyde for 20 min and treated with 0.5% Triton X-100 for 10 min at room temperature. Then, the cells were stained with Click Reaction mix for 30 min, and the nuclei were stained using DAPI. Proliferation rate was determined by quantifying the percentage of EdU^+^ cells using fluorescence microscope.

### Transwell-Migration Assay

8μm transwell inserts (Falcon, USA) were used to measure the migratory of cells. Briefly, 5×10^4^ HCC cells in 200 μl of DMEM were placed into the upper chamber, 500 μl DMEM medium containing 10% FBS was added to the lower chambers. After 48 h, the non-migrated cells on the top side were wiped carefully and the inserts were fixed in 5% formaldehyde solution for 10 min. After that, each inset was stained with 0.1% crystal violet for 10 min, then washed with PBS for three times. Three random microscopic fields were captured and cells were counted for each group.

### Cell Apoptosis and Cell Cycle Assay

Stable 97H and Huh7 cells were seeded into 6-well plates at a density of 5× 10^5^ cells per well 24 hours before the apoptosis assay. After treating with 20 μM cisplatin (Selleck Chemicals, S1166) for 24 hours, cells were harvested by trypsinization, washed twice with PBS, stained with APC-Annexin V and PI following the manufacturers’ instructions (MultiSciences, 70-AP107-100). Flow cytometry was performed using Beckman CytoFLEX and the results were analyzed with FlowJo software.

For cell cycle analysis, cells were harvested and fixed in cold 70% ethanol overnight at 4°C. Cells were then stained with PI staining solution (Sangon Biotech, E607306) for 15min, followed by washing with PBS twice. Flow cytometry was performed using Beckman CytoFLEX and the results were analyzed with FlowJo software.

### Intracellular ROS Assay

The intracellular ROS level was detected by CellROX™ Cytometry Assay Kit (Thermo Fisher, C10493). Briefly, cells were harvested and stained with 500 nM CellRox reagent for 60 min at 37°C. After washing the cells once with PBS, immediately analyzed the samples by Beckman CytoFLEX. The results were analyzed with FlowJo software.

### Seahorse XF Cell Energy Phenotype Assay

Cellular energy phenotypes and metabolic switching was measured by Seahorse XFe96 Analyzer (Agilent, USA) with Seahorse XF Cell Energy Phenotype Test Kit (Agilent, 103325-100). In brief, 0.5-1 × 10^5^ cells were seeded in 96-well Seahorse plates with XF RPMI medium supplemented with 2 mM glutamine, 10 mM glucose, 1mM pyruvate, and 5 mM HEPES, and then incubated in a CO_2_-free incubator for 1 hour prior to the assay. For assessment of energy phenotypes, 100 μM oligomycin and FCCP were used according to the manufacturer’s instructions.

### β-Galactosidase Staining

The β-galactosidase staining was performed using Senescence β-Galactosidase Staining Kit (Beyotime, C0602) according to the manufacturer’s instructions. Briefly, 5×10^5^ cells were planted in 6-well plates with coverslips 24 hours before experiments. The cells were fixed using 4% paraformaldehyde for 20 min and then stained with working solution at 37°C overnight. The second day, cells were washed with PBS for three times and observed under microscope. Senescence rate was determined by quantifying the percentage of positive cells.

### ATP and Malondialdehyde (MDA) Assay

To measure the intracellular ATP level, we utilized the CellTiter-Glo Cell Viability Assay (Premega, G9241). Control and stable *ABCC6* knockdown HCC cells were seeded into 96-well plates at a density of 1 × 10^4^ cells per well and incubated at 37°C. The second day, added 100μl CellTiter-Glo reagent to the medium in each well, mixed the contents for 2 minutes and incubated at room temperature for 10 minutes. The luminescent signals were measured using a SpectraMax i3 microplage reader (Molecular Devices, USA).

To evaluate lipid peroxidation, we utilized the MDA assay (Beyotime, S0131S). Control and stable *ABCC6* knockdown HCC cells were seeded into 6-well plates at a density of 1 × 10^6^ cells per well and incubated at 37°C. Cells were lysed using Western lysis buffer, and the protein concentration was measured using a BCA protein Assay kit (Thermo Fisher). MDA assay was performed according to the manufacturer’s instructions. Signal was measured at OD 532 nm using a SpectraMax i3 microplage reader (Molecular Devices, USA). Relative MDA level was normalized by the protein concentration of each sample.

### *In Vivo* Tumor Growth Assays

NSG (NOD-*scid* IL2Rgamma^null^) mice were purchased from Shanghai Model Organisms Ltd. and bred at the specific-pathogen-free core facilities in Renji Hospital. All mice were housed on a 12 hours-12 hours light-dark cycle. 5×10^6^ shNS and sh*ABCC6* 97H cells were injected subcutaneously into 6-weeks-old NSG mice (n=4 per group). Tumors were measured using a caliper and the tumor volume was calculated as (width × width × length/2). Mice were euthanized when they met the institutional euthanasia criteria for tumor size. At the end-point, tumors were collected and weighted. Animal experimental protocols were approved by the Institutional Animal Care and Use Committee of Renji Hospital, Shanghai Jiao Tong University School of Medicine.

### Western Blot

Total protein was extracted using RIPA Lysis Buffer (Thermo Fisher) according to the manufacturer’s instructions. The protein concentration was measured using a BCA protein Assay kit (Thermo Fisher). Equal amounts of proteins were separated by 8-10% SDS-PAGE gel and then transferred onto polyvinylidene fluoride membranes. Chemiluminescence signaling was detected by ECL Western Blotting Substrate (Vazyme, E412-01). Antibodies used for western blot were listed below: ABCC6 (Proteintech, 27848-1-AP), β-actin (Cell Signaling Technology, 3700S), PPARα (Novus, NB300-57), Cyclin D1 (Cell Signaling Technology, 2978S), ACOX1 (Proteintech, 10957-1-AP).

### Real-Time Quantitative PCR

Total RNA was extracted from cell liens using Trizol (Thermo Fisher) according to the manufacturer’s instructions. RNA concentration was measured using Thermo Scientific Nanodrop Spectrophotometer. 0.8-1 µg total RNA was subjected to reverse transcription using HiScript III 1st Strand cDNA Synthesis Kit (Vazyme, R312-01) according to the manufacturer’s instructions. The Quantitative real-time PCR (qRT-PCR) was conducted using ChamQ SYBR Color qPCR Master Mix (Vazyme, Q441-02) in the Bio-Rad Real-Time PCR system. Gene expression levels were normalized to the expression of *GAPDH* or *ACTB*. Primers used for qRT-PCR were listed below: ABCC6-F: AGATGGTGCTTGGATTCGCC, ABCC6-R: GCCACACAGTAGGATGAATGAG; ABCA6-F: AAACAGAAAAGCGTGTATCAGCA, ABCA6-R: GAGGAGCCATTCCAGGAAACT; ABCG5-F: TGGACCAGGCAGATCCTCAAA, ABCG5-R: CCGTTCACATACACCTCCCC; PPARA-F: TTCGCAATCCATCGGCGAG, PPARA-R: CCACAGGATAAGTCACCGAGG; ACOX1-F: GGAACTCACCTTCGAGGCTTG, ACOX1-R: TTCCCCTTAGTGATGAGCTGG; CCND1-F: GCTGCGAAGTGGAAACCATC, CCND1-R: CCTCCTTCTGCACACATTTGAA; GAPDH-F: GGAGCGAGATCCCTCCAAAAT, GAPDH-R: GGCTGTTGTCATACTTCTCATGG.

### RNA-Seq

Total RNA was isolated from shNS and sh*ABCC6* 97H cells using Trizol (Thermo Fisher) according to the manufacturer’s instructions. rRNA was subsequently depleted from total RNA using NEBNext rRNA depletion kit (New England BioLabs). After that, NEBNext Ultra Directional RNA Library Prep Kit (New England BioLabs) was used for library preparation. RNA sequencing was performed by Annorad Ltd. using Novaseq 6000 platform. GO and KEGG analysis were performed using David Bioinformatics Resources 6.8 (https://david.ncifcrf.gov/). Gene set enrichment analysis (GSEA) was carried out using GSEA software v2.0.

### Statistical Analysis

Data are presented as mean ± standard error of the mean (SEM). Statistical comparisons were performed by using two-tailed t-tests, one-way ANOVA or Kaplan-Meier analysis. *P* values less than 0.05 were considered statistically significant. NS, not significant. All statistical analyses were carried out using R (Version 4.0.1) or Graphpad Prism 8 (GraphPad Software).

## Results

### ABC Transporter Family Gene Expression and Behavior in HCC

**Figure 1:**
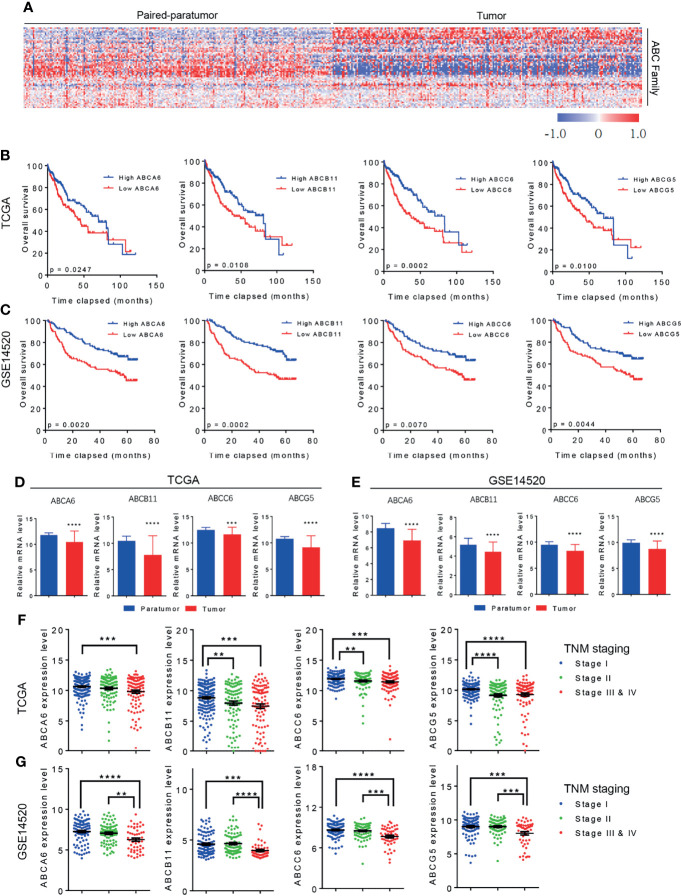
The Role of ABC transporter family genes in HCC. **(A)** Heat map showing the differential expression of ABC transporter family genes in TCGA dataset. **(B)** Kaplan-Meier analysis showing the overall survival of patients with HCC in correlation with high or low ABC family genes level. Data was derived from TCGA dataset. **(C)** Kaplan-Meier analysis showing the overall survival of HCC patients in correlation with high or low ABC family genes level. Data was derived from GSE14520 dataset. **(D)** TCGA dataset showing the differential expression of ABC family genes in HCC paratumor tissues and tumor tissues. **(E)** GSE14520 dataset showing the differential expression of ABC family genes in HCC paratumor tissues and tumor tissues. **(F)** TCGA database showing the expression level of ABC family genes with advanced HCC TNM staging. **(G)** GSE14520 dataset showing the expression level of ABC family genes with advanced HCC TNM staging. Error bars indicate means ± SEM. *P*-values were determined by two tailed *t*-test **(D, E)**, one-way ANOVA **(F, G)** and Kaplan-Meier analysis **(B, C)**. **P < 0.01, ***P < 0.001, ****P < 0.0001.

**Figure 2:**
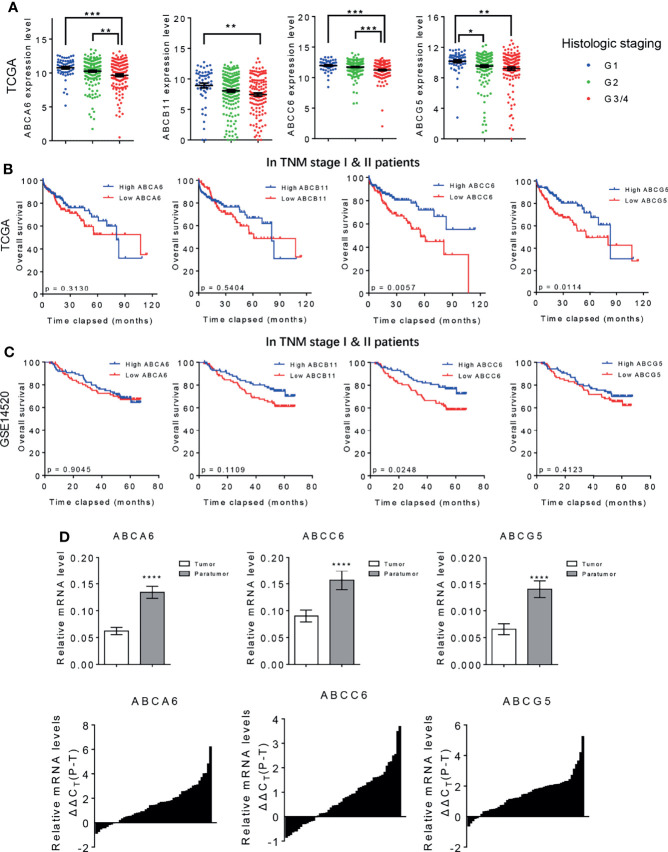
*ABCA6*, *ABCC6*, and *ABCG5* might serve as tumor suppressor genes in HCC. **(A)** TCGA database showing the expression level of ABC family genes in different HCC histologic staging. **(B)** Kaplan-Meier analysis showing the overall survival of early stage HCC patients (TNM I & II) with high or low ABC family genes level. Data was derived from TCGA database. **(C)** Kaplan-Meier analysis showing the overall survival of early stage HCC patients (TNM I & II) with high or low ABC family genes level. Data was derived from GSE14520 dataset. **(D)** qPCR analysis validating the differential expression of *ABCA6*, *ABCC6* and *ABCG5* in 50 pairs of early-stage HCC tumor and paratumor tissues. Error bars indicate means ± SEM. *P*-values were determined by two tailed *t*-test **(D)**, one-way ANOVA **(A)** and Kaplan-Meier analysis **(B, C)**. *P < 0.05, **P < 0.01, ***P < 0.001, ****P < 0.0001.

To evaluate the role of ABC family genes in HCC, we first analyzed two public HCC datasets; the Cancer Genome Atlas (TCGA) and the GSE14520 (with the largest number of HCC patients and detailed clinical information) datasets. By comparing the expression levels of ABC family genes in HCC tumors and paratumors, we found that approximately 1/3 of the ABC genes were significantly upregulated, and 1/3 were downregulated (**Figure 1A**). After incorporating the clinical prognosis information into analysis, it was noted that some ABC family genes correlated with overall survival or progression-free survival of patients HCC. However, only *ABCA6*, *ABCB11*, *ABCC6*, and *ABCG5* showed consistent results in both TCGA and GSE14520 datasets (**Tables 1**, **2**). Notably, high *ABCA6*, *ABCB11*, *ABCC6*, and *ABCG5* expression levels were correlated with favorable overall survival (**Figures 1B, C**). Additionally, bioinformatics analyses also showed that the mRNA levels of these genes were attenuated in tumors comparing with those in paratumor tissues (**Figures 1D, E**). Furthermore, expression levels of these genes gradually decreased as TNM staging advanced in both TCGA and GSE14520 datasets (**Figures 1F, G**). Similarly, we also found that their expression levels decreased with advanced HCC histological staging in the TCGA dataset (**Figure 2A**). Collectively, our data suggest that among all ABC family genes, *ABCA6*, *ABCB11*, *ABCC6*, and *ABCG5* might serve as potential tumor suppressor genes in HCC.

**Table 1:** Cox regression analysis of ABC family genes in GSE14520 cohort.

GSE14520 cohort (n=242)					
Factor	OS		Factor	PFS	
	Relative hazard (95% CI)	*P*		Relative hazard (95% CI)	*P*
Low ABCA6 expression	1.884 (1.253 to 2.834)	0.002	Low ABCB11 expression	1.620 (1.153 to 2.277)	0.005
Low ABCB11 expression	2.191 (1.442 to 3.331)	<0.001	High ABCD3 expression	1.437 (1.024 to 2.017)	0.036
Low ABCB4 expression	1.606 (1.072 to 2.405)	0.022	Low ABCG5 expression	1.484 (1.058 to 2.081)	0.022
Low ABCC6 expression	1.739 (1.157 to 2.615)	0.008			
High ABCD3 expression	1.666 (1.108 to 2.505)	0.014			
Low ABCG5 expression	1.797 (1.193 to 2.706)	0.005			

**Table 2:** Cox regression analysis of ABC family genes in TCGA cohort.

TCGA cohort (n=365)							
Factor	OS		Factor	PFS			
	Relative hazard (95% CI)	*P*		Relative hazard (95% CI)			*P*
Low ABCA6 expression	1.433 (1.012 to 2.028)	0.043	High ABCA2 expression		1.481 (1.060 to 2.070)	0.021	
Low ABCA8 expression	1.488 (1.048 to 2.113)	0.026	Low ABCA9 expression		1.493 (1.070 to 2.084)	0.018	
Low ABCA9 expression	1.794 (1.255 to 2.564)	0.001	Low ABCD2 expression		1.845 (1.313 to 2.591)	<0.001	
Low ABCB11 expression	1.463 (1.033 to 2.071)	0.032	High ABCF2 expression		1.615 (1.152 to 2.265)	0.005	
High ABCB6 expression	1.470 (1.036 to 2.085)	0.031					
High ABCC1 expression	1.706 (1.201 to 2.425)	0.003					
High ABCC5 expression	1.587 (1.121 to 2.248)	0.009					
Low ABCC6 expression	2.044 (1.432 to 2.918)	<0.001					
High ABCC8 expression	1.419 (1.001 to 2.011)	0.049					
Low ABCD2 expression	1.586 (1.117 to 2.252)	0.010					
High ABCF2 expression	1.473 (1.037 to 2.092)	0.030					
Low ABCG5 expression	1.561 (1.098 to 2.218)	0.013					
Low ABCG8 expression	1.735 (1.218 to 2.473)	0.002					

### Identification of *ABCC6* as a Biomarker for Early-Stage HCC

It is well-established that overall survival rates are significantly higher in patients with HCC that are diagnosed at an early-stage and receive immediate treatment (21). However, because early-stage HCC does not present significant symptoms, delayed diagnosis and treatment are common and likely contribute to poor cancer outcomes (22). To further assess the clinical value of *ABCA6*, *ABCB11*, *ABCC6*, and *ABCG5* in patients with HCC, we re-analyzed the public datasets and focused on early-stage HCC (TNM stage I & II). Interestingly, Kaplan-Meier analyses showed that *ABCC6* positively correlates with overall survival in TNM stage I and II HCC patients in both datasets, suggesting that *ABCC6* displays significant prognostic value in early-stage HCC. In contrast, *ABCA6*, *ABCB11*, and *ABCG5* did not display consistent prognostic value in early-stage HCC (**Figures 2B, C**).

Furthermore, we measured the expression of *ABCA6*, *ABCC6*, and *ABCG5* in 50 pairs of early-stage tumor tissues and paratumor tissues using quantitative reverse transcription polymerase chain reaction (qRT-PCR). Consistent with our bioinformatic analysis, this assessment also showed significantly lower *ABCC6* expression in tumor tissues than in paratumor tissues (**Figure 2D**), suggesting that *ABCC6* might be a potential diagnostic marker in HCC.

### Evaluating the Clinical Effects of ABCC6 Expression in Patients With HCC

**Figure 3:**
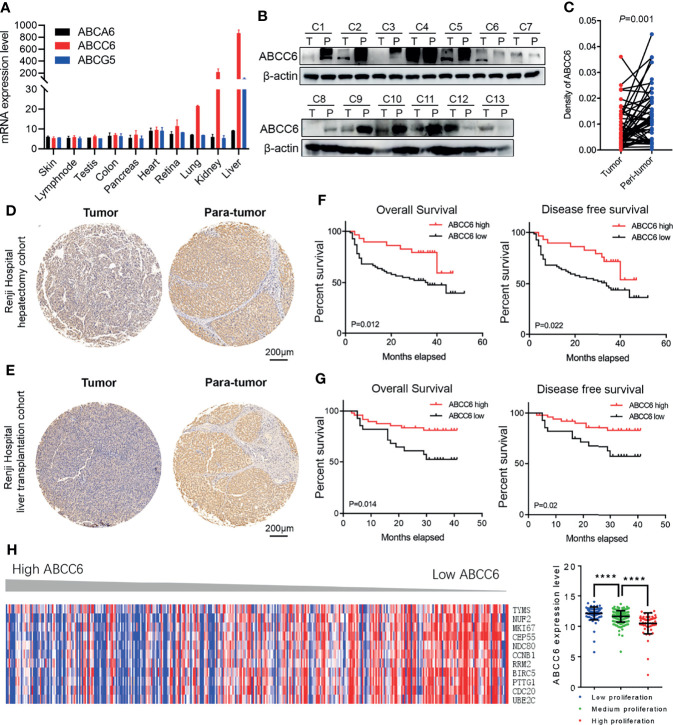
The clinical outcomes of ABCC6 in patients with HCC. **(A)** BioGPS database showing the mRNA expression level of *ABCA6*, *ABCC6*, and *ABCG5* in different normal human tissues. **(B)** Western blotting displaying ABCC6 protein levels in 13 pairs of HCC tumor and paratumor tissues. **(C)** Statistical analysis showing the differential protein level of ABCC6 in Renji Hospital hepatectomy HCC tissue array. **(D)** Representative IHC figure showing the ABCC6 protein level in Renji Hospital hepatectomy HCC tissue array. **(E)** Representative IHC figure showing the ABCC6 protein level in Renji Hospital liver transplantation HCC tissue array. **(F)** Kaplan-Meier analysis showing the overall survival and disease-free survival of HCC patients in correlation with high or low *ABCC6* levels in Renji Hospital hepatectomy cohort. **(G)** Kaplan-Meier analysis showing the overall survival and disease-free survival of HCC patients in correlation with high or low *ABCC6* levels in Renji Hospital liver transplantation cohort. **(H)** Heatmap and histogram showing the expression of proliferation-related genes in correlation with different *ABCC6* expression level. Data was derived from TCGA dataset. Error bars indicate means ± SEM. *P*-values were determined by two tailed *t*-test **(C)**, one-way ANOVA **(H)** and Kaplan-Meier analysis **(F, G)**. ****P < 0.0001.

Our previous studies have identified *ABCA6*, *ABCC6* and *ABCG5* as potential tumor-suppressor genes in HCC. To further narrow down our candidate gene, we assessed the mRNA level of these genes in normal human tissues using the BioGPS database (http://biogps.org). Only *ABCC6* was found to be specifically expressed in normal liver tissue (**Figure 3A**), highlighting its importance in liver homeostasis. Moreover, western blotting assays confirmed lower ABCC6 expression in tumor tissue than in paratumor tissues (**Figure 3B**). Accordingly, we focused on the function of ABCC6 in the development and progression of HCC.

To further validate our bioinformatic data, we employed two independent HCC cohorts. Cohort 1 (hepatectomy cohort) included 153 individuals who were hospitalized between 2010 and 2015 who underwent liver resection surgery at the Department of Liver Surgery, Renji Hospital, Shanghai Jiao Tong University School of Medicine. Cohort 2 (liver transplantation cohort) included 70 individuals who underwent liver transplantation between 2015 and 2016 at the Department of Liver Surgery, Renji Hospital, Shanghai Jiao Tong University School of Medicine. To evaluate the association between ABCC6 expression and clinicopathological features of HCC, we used immunohistochemistry to detect ABCC6 expression in tissue microarrays of patients from both cohorts. Consistently, ABCC6 was significantly downregulated in tumor tissues compared to paratumor tissues (**Figures 3C–E**). We divided all cases into high and low ABCC6 groups according to the immunohistochemistry expression level, and noticed that patients with high ABCC6 expression displayed increased overall survival and disease-free survival (**Figures 3F, G**), which is consistent with the TCGA and GSE14520 bioinformatic results. Multivariate Cox regression analysis of clinical prognostic information demonstrated that downregulated ABCC6 was not only an independent prognostic marker, but also correlated with some malignant and aggressive clinicopathological features, such as high AFP values and large tumor size (**Table 3**). Furthermore, we interrogated the TCGA database and analyzed the relationship between *ABCC6* expression and several proliferation markers (e.g., *MKI67*, *CCNB1*, *RRM2*, and *CDC20*). A negative correlation was observed between expression of *ABCC6* and these proliferation markers (**Figure 3H**). Collectively, our data suggest that ABCC6 is downregulated in HCC tumor tissues and correlates with favorable outcomes in patients with HCC.

**Table 3:** Cox regression analysis of risk factors associated with overall survival in Renji Hospital hepatectomy cohort.

Variables	Univariate analysis			Multivariate analysis		
	HR	95% CI	*P* Value	HR	95% CI	*P* Value
ABCC6 expression (High vs. Low)	0.33	0.14-0.78	0.012	0.33	0.14-0.78	0.012
Gender (Male vs. Female)	1.65	0.74-3.72	0.225			
Age (≧50 vs.<50)	0.97	0.52-1.81	0.991			
AFP (≧200 vs.<200)	2.78	1.44-5.33	0.002	1.84	0.93-3.63	0.049
ALT (≧50 vs.<50)	1.82	0.98-3.40	0.058			
Tumor size (≧5 cm vs.<5cm)	2.75	1.44-5.23	0.002	2.18	1.10-4.34	0.026
Tumor nodule number (Multiple vs. Single)	0.50	0.27-0.93	0.027			
Cancer embolus (Presence vs. Absence)	8.18	4.21-15.90	<0.001	6.14	3.04-12.42	<0.001
Pathology grade	1.34	0.74-2.44	0.334			
Tumor necrosis (Presence vs. Absence)	2.36	1.04-5.32	0.039			
Cirrhosis (Presence vs. Absence)	2.41	0.95-6.13	0.066			

### *ABCC6* Knockdown Promotes HCC Proliferation *In Vitro* and *In Vivo*

**Figure 4:**
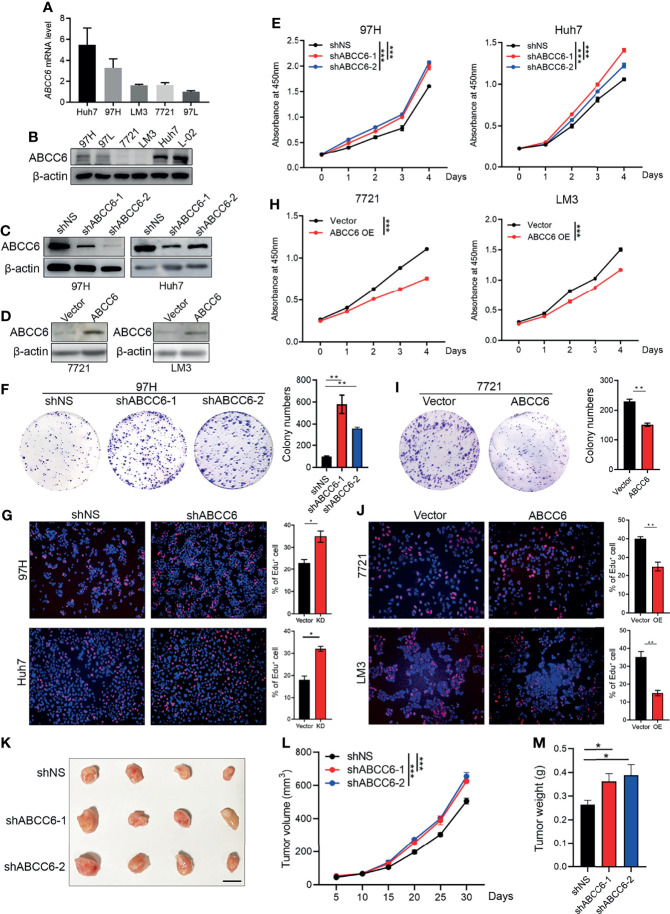
*ABCC6* knockdown promotes HCC cell lines proliferation *in vitro* and *in vivo*. **(A)** qPCR analysis showing *ABCC6* mRNA expression level in different HCC cell lines. **(B)** Western blot displaying ABCC6 protein level in different HCC cell lines as well as one normal human hepatic cell line (L-02). **(C)** Western Blot validating the ABCC6 knockdown efficiency in 97H and Huh7 cell lines. **(D)** Western Blot validating the ABCC6 overexpression efficiency in 7721 and LM3 cell lines. **(E)** CCK-8 assay showing the cell proliferation of *ABCC6* knockdown 97H and Huh7 cells. **(F)** Colony formation assay of *ABCC6* knockdown 97H cells. **(G)** Edu incorporation assay showing the effect of *ABCC6* knockdown on the cell proliferative ability of 97H and Huh7 cells. **(H)** CCK-8 assay showing the cell proliferation of *ABCC6* overexpression 7721 and LM3 cells. **(I)** Colony formation assay of *ABCC6* overexpression 7721 cells. **(J)** Edu incorporation assay showing the effect of *ABCC6* overexpression on the cell proliferative ability of 7721 and LM3 cells. **(K)** Images showing the subcutaneous xenograft of ShNS, ShABCC6-1, and ShABCC6-2 97H cells. n=4; The scale bar indicates 1cm. **(L)** The growth curves of ShNS, ShABCC6-1, and ShABCC6-2 97H cells subcutaneously injected into NSG immunodeficient mice. (M) The weight of tumors collected from xenograft NSG mouse model implanted with ShNS, ShABCC6-1, and ShABCC6-2 97H cells. Error bars indicate means ± SEM. *P*-values were determined by two tailed *t*-test **(E–J)** and one-way ANOVA **(L, M)**. *P < 0.05, **P < 0.01, ***P < 0.001.

Next, we investigated the effect of ABCC6 on the biological function of HCC cell lines *in vitro*. ABCC6 expression and protein levels were first examined in five commercial available HCC cell lines (MHCC97H, MHCC97L, SMMC7721, Huh7, and HCC-LM3) and one normal human hepatic cell line (L-02). Enhanced ABCC6 levels were observed in MHCC97H, MHCC97L, and Huh7 cells, while lower ABCC6 levels were observed in SMMC7721 and HCC-LM3 cells. All HCC cell lines displayed lower ABCC6 protein levels than normal hepatic cell line (**Figures 4A, B**). Furthermore, different HCC cell lines were chosen for loss- or gain-of-function studies based on their endogenous ABCC6 levels. *ABCC6* knockdown (using two short hairpin RNAs) or overexpression efficiency was confirmed by western blotting (**Figures 4C, D**). CCK-8 assay, colony formation assay and EdU (5-Ethynyl-2’-Deoxyuridine) incorporation assays all showed significantly enhanced proliferation ability in HCC cells with ABCC6 knockdown (**Figures 4E–G**), and the proliferation ability was decreased after ABCC6 overexpression (**Figures 4H–J**). Taken together, these data suggest that ABCC6 plays a tumor-suppressive role by inhibiting HCC proliferation *in vitro*.

We further examined the role of ABCC6 in HCC development and progression *in vivo* using an NSG (NOD-*scid* IL2Rgamma^null^) xenograft mouse model. The same number of control or *ABCC6* knockdown MHCC97H cells was injected subcutaneously into the left flank of NSG mice. As expected, tumor size, tumor growth curve, and tumor weight were substantially augmented following *ABCC6* knockdown (**Figures 4K–M**), indicating that *ABCC6* depletion also facilitates tumor progression *in vivo*.

### *ABCC6* Knockdown Influences Cell Cycle and Cell Apoptosis

**Figure 5:**
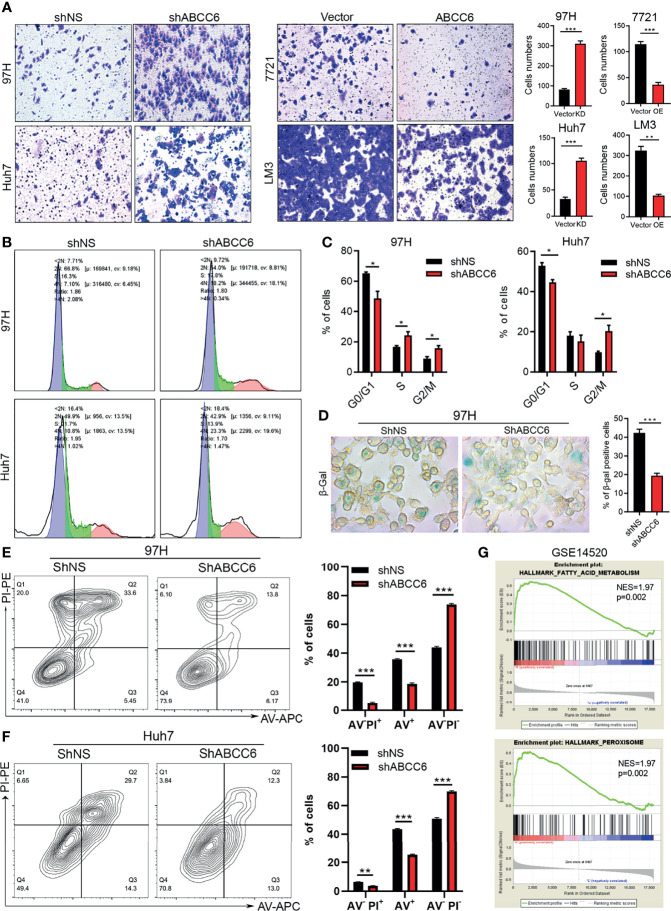
*ABCC6* affect HCC cell lines migration, cell cycle arrest and apoptosis. **(A)** Transwell assay showing the effect of *ABCC6* knockdown and overexpression in the migration ability of HCC cell lines. **(B)** The results of cell cycle analysis by flow cytometry in *ABCC6* knockdown 97H and Huh7 cells. The blue peak and the red peak represents G0-G1, G2-M stage respectively. The middle is S stage. **(C)** Statistical analysis showing the effect of *ABCC6* knockdown in the cell cycle of 97H and Huh7 cells. **(D)** β-galactosidase staining showing the senescence status of HCC cell lines after *ABCC6* knockdown. **(E)** Flow cytometry analysis showing the effect of *ABCC6* knockdown on the apoptosis of 97H cells treating with 20 μM cisplatin for 24 hours. **(F)** Flow cytometry analysis showing the effect of *ABCC6* knockdown on the apoptosis of Huh7 cells treating with 20 μM cisplatin for 24 hours. **(G)** GSEA analysis showing the enrichment pathway of *ABCC6* high samples in GSE14520 dataset. Error bars indicate means ± SEM. *P*-values were determined by two tailed *t*-test **(A, C–F)**. *P < 0.05, **P < 0.01, ***P < 0.001.

To further study the role of ABCC6 in tumor biology, we knocked down or overexpressed *ABCC6* in HCC cells and evaluated their migration ability. The results showed that migrative ability was promoted by *ABCC6* depletion and hindered by *ABCC6* overexpression (**Figure 5A**). In addition, cell cycle analysis showed that *ABCC6* knockdown significantly increased the percentage of cells in the G2/M phase and decreased the G0/G1 phase, suggesting that ABCC6 may regulate the cell cycle in cancer cells (**Figures 5B, C**). *ABCC6* depletion also affected the proportion of β gal-positive cells *in vitro* (**Figure 5D**), revealing the possible role of ABCC6 in regulating cell senescence. Furthermore, *ABCC6* knockdown significantly inhibited cisplatin-induced cell apoptosis in both 97H and Huh7 cells (**Figures 5E, F**), implying that *ABCC6* depletion might contribute to HCC growth by ensuring apoptosis resistance.

**Figure 6:**
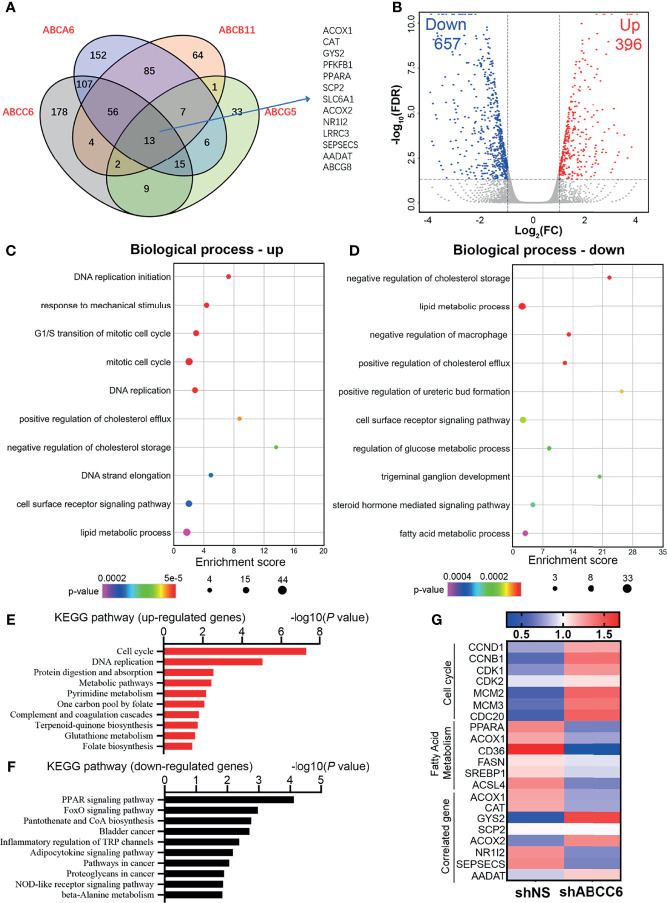
Transcriptome-wide analysis of *ABCC6* knockdown 97H cells. **(A)** Venn diagram showing the overlap among positive correlation (R>0.4) genes of *ABCA6*, *ABCB11*, *ABCC6* and *ABCG5*. Data was derived from TCGA database. **(B)** Volcano plot showing the up-regulated and down-regulated genes in *ABCC6* knockdown 97H cells. Cut-off value: |log_2_(FC)|>1, *P*<0.05. **(C)** Gene Ontology (GO) enrichment analysis of up-regulated genes in *ABCC6* knockdown 97H cells. **(D)** Gene Ontology (GO) enrichment analysis of down-regulated genes in *ABCC6* knockdown 97H cells. **(E)** KEGG pathway analysis of up-regulated genes in *ABCC6* knockdown 97H cells. **(F)** KEGG pathway analysis of down-regulated genes in *ABCC6* knockdown 97H cells. **(G)** Heatmap showing the relative expression levels of cell cycle, fatty acid metabolism and *ABCC6* correlated genes based on the RNA-seq results.

We then performed gene set enrichment analysis (GSEA) using GSE14520 sequence data. Notably, the “fatty acid metabolism” and “peroxisome” pathways were highly active in samples with high expression of *ABCC6* (**Figure 5G**). Furthermore, to evaluate the potential down-stream targets of ABC family genes, we analyzed the TCGA dataset and overlapped the co-expression gene (R>0.4 or <-0.4) of *ABCC6*, *ABCA6*, *ABCB11*, *ABCG5*. Thirteen genes were identified as co-expression genes through overlapping (**Figure 6A**). Intriguingly, *CAT*, *PPARA*, *ACOX1*, and *ACOX2* are essential peroxisomal genes.

### Transcriptome-Wide Analysis of ABCC6 in HCC Cells

Next, we sought to elucidate the mechanism by which ABCC6 regulates HCC cell proliferation. To comprehensively assess the effects of ABCC6 on HCC cells, we performed RNA-sequencing using control and *ABCC6*-knockdown MHCC97H cells. The volcano plot showed that 657 genes were downregulated while 396 genes were upregulated after *ABCC6* depletion (|log2(FC)|>1, *P*<0.05) (**Figure 6B** and **Supplementary Table 1**). Gene Ontology (GO) analysis showed that “DNA replication” and “cell cycle” pathways were significantly enhanced as a result of *ABCC6* knockdown, while lipid and fatty acid metabolism were significantly inhibited (**Figures 6C, D**). Additionally, the Kyoto Encyclopedia of Genes and Genomes (KEGG) analysis also exhibited the upregulation of “cell cycle” and “DNA replication” pathways, and the downregulation of “PPAR signaling pathway” (**Figures 6E, F**). Finally, we examined the expression of key genes in the “cell cycle” and “fatty acid metabolism” pathways. Strikingly, the expression of *CCND1*, *CCNB1*, *CDK1*, and *CDK2* was all significantly augmented by *ABCC6* knockdown, while *PPARA*, *ACOX1*, *CD36*, *ACSL4* were mitigated (**Figure 6G**). Taken together, these data suggest that *ABCC6* depletion enhances cell proliferation by regulating peroxisome activity.

### *ABCC6* Knockdown Inhibits PPARα Activity

Peroxisomes are membrane-bound oxidative organelles found in the cytoplasm and involve more than 50 different metabolic enzymes. Peroxisomes play key roles in lipid metabolism and are essential for ROS production (23). Recent studies have reported that cancer cells exhibit remarkable alterations in peroxisome activity (24), and peroxisome metabolism is largely reduced in HCC (25). Our previous results demonstrated that *ABCC6* is positively correlated with both “peroxisome” pathway and essential peroxisomal genes in the HCC public dataset; *ABCC6* knockdown significantly inhibited the expression of *PPARA* and *ACOX1* in HCC cells. Thus, we consider the possibility whether *ABCC6* depletion promotes cell proliferation by inhibiting peroxisome activity.

**Figure 7:**
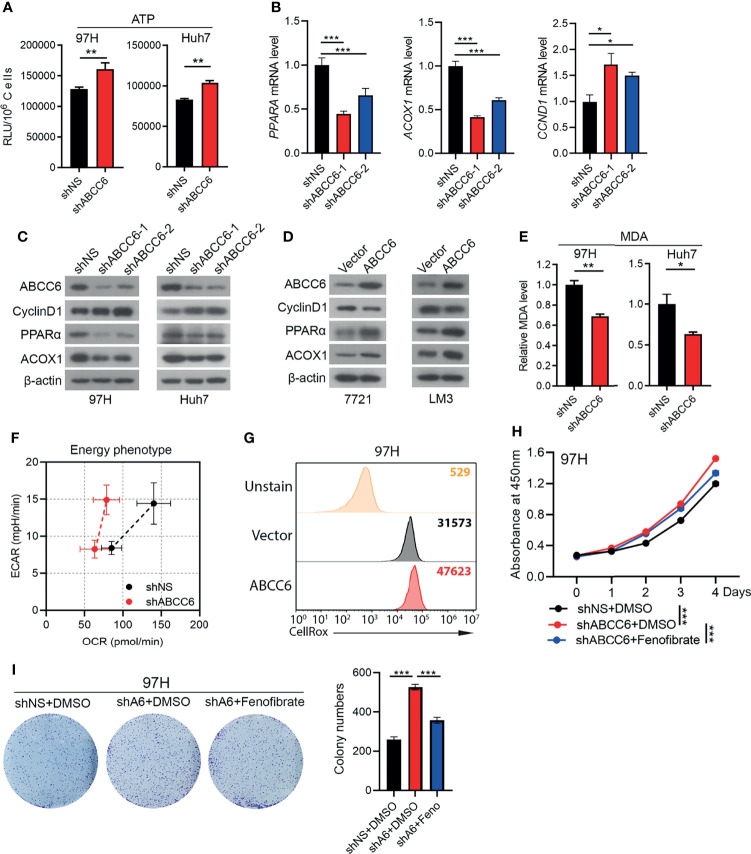
*ABCC6* knockdown suppressed PPARα expression in HCC cells. **(A)** ATP level of *ABCC6* knockdown and control 97H and Huh7 cells. **(B)** qPCR analysis showing the expression of *PPARA*, *ACOX1* and *CCND1* in *ABCC6* knockdown 97H cells. **(C)** Western blot showing the protein levels of ABCC6, Cyclin D1, PPARα and ACOX1 in *ABCC6* knockdown 97H and Huh7 cells. **(D)** Western blot showing the protein levels of ABCC6, Cyclin D1, PPARα and ACOX1 in *ABCC6* overexpression 7721 and LM3 cells. **(E)** MDA assay showing the relative lipid peroxidation level of *ABCC6* knockdown and control 97H and Huh7 cells. **(F)** Seahorse assay displaying the baseline and stressed energy phenotype of *ABCC6* knockdown and control 97H cells. **(G)** Flow cytometry analysis showing the intracellular ROS level of vector and *ABCC6* overexpression 97H cells. **(H)** CCK-8 assay showing the effect of PPARα agonist fenofibrate in the proliferation of *ABCC6* knockdown and control 97H cells. **(I)** Colony formation assay showing the effect of PPARα agonist fenofibrate in the proliferation of *ABCC6* knockdown and control 97H cells. Error bars indicate means ± SEM. *P*-values were determined by two tailed *t*-test **(A, E)** and one-way ANOVA **(B, H, I)**. *P < 0.05, **P < 0.01, ***P < 0.001.

It is well-established that *ABCC6* overexpression results in the efflux of ATP, which decreases intracellular ATP levels. Accordingly, we found that *ABCC6* knockdown increased intracellular ATP levels (**Figure 7A**). Consistent with our RNA-sequence results, qRT-PCR analysis showed that *PPARA* and *ACOX1* were decreased after *ABCC6* depletion, while the mRNA level of *CCND1* was enhanced (**Figure 7B**). Furthermore, western blotting indicated that *ABCC6* knockdown mitigated the protein levels of PPARα and ACOX1, while enhanced the protein level of CyclinD1, vice versa (**Figures 7C, D**). As PPARα and ACOX1 mainly influence lipid metabolism, we performed the malondialdehyde (MDA) assay to evaluate lipid peroxidation. As expected, *ABCC6* knockdown significantly inhibited lipid peroxidation in HCC cells (**Figure 7E**). Moreover, seahorse assay showed that *ABCC6* depletion affected the cellular energy phenotype and mitigated the oxygen consumption rate (OCR) of cancer cells under stress conditions (**Figure 7F**), most likely due to the lower lipid peroxidation activity. We further examined the intracellular ROS levels and found that *ABCC6* overexpression elevated ROS levels (**Figure 7G**). To determine whether PPARα directly contributes to cell proliferation, we treated *ABCC6* knockdown cells with PPARα agonist fenofibrate and monitored cell growth. Consistently, fenofibrate treatment partially rescued the cell proliferation phenotypes (**Figures 7H, I**). Taken together, these findings support the notion that *ABCC6* knockdown inhibits peroxisomal ACOX1 and PPARα to prevent oxidative damage in HCC cells, thereby enhancing cell proliferation.

## Discussion

ABC transporter family genes have diverse roles in energy homeostasis, lipid metabolism, drug resistance, and tumor progression. Although several studies suggested that ABC transporter family genes might serve as cancer drivers in multiple cancer types (7), other studies indicated that some ABC family genes may be tumor suppressors. For example, eight ABC transporter genes (e.g., *ABCA8*, *ABCC6*, and *ABCC9*) were significantly downregulated in prostate cancer tissues compared with noncancerous tissues (26). However, the behavior of ABC transporter family genes in HCC has not been clearly investigated. In this study, we evaluated the expression level and prognostic prediction value of all ABC transporter family members in TCGA and GSE14520 datasets, and found that ABCC6 is significantly downregulated in HCC tumor tissues and correlated with favorable outcomes in patients with HCC. Consistent with our findings, a previous study also demonstrated that the ABCC6 protein was highly expressed in liver tissue, but remained non-detectable in a small panel of human tumor samples (27).

It is well-established that *ABCC6* mutation is responsible for PXE; however, the biological function of ABCC6 in HCC was largely unexplored. Our *in vitro* and *in vivo* results indicated that *ABCC6* knockdown significantly promoted HCC cell proliferation. Moreover, *ABCC6* depletion suppressed cell cycle arrest and apoptosis. To further investigate the mechanisms of ABCC6 in HCC, we performed GSEA, co-correlation analysis, and transcriptome sequencing. Notably, GO and KEGG analysis indicated that “cell cycle” and “DNA replication” pathways were significantly upregulated, while “PPAR signaling pathway” was downregulated. Furthermore, *PPARA* and *ACOX1* were found to be potential targets of ABCC6.

PPARα, a key regulator of peroxisome metabolism, is closely related to energy homeostasis and lipid metabolism (28). Recent studies have shown that PPARα-deficient mice are remarkably sensitive to DEN(diethylnitrosamine)-induced liver cancer, and PPARα inhibits HCC development by mediating the NF-κB pathway (29). Additionally, the PPARα agonist, fenofibrate, caused inhibitory effects in different cancer cell lines and animal tumor models, including hepatocarcinogenesis (30–32). In our study, we found that *ABCC6* knockdown suppressed PPARα expression transcriptionally, and fenofibrate partially rescued the cell proliferation phenotype, suggesting that PPARα is the downstream target of ABCC6. Moreover, ACOX1 is the rate-limiting enzyme in fatty acid β-oxidation. A previous study suggested that ACOX1 is controlled by PPARα, and ACOX1 dysfunction contributes to hepatocarcinogenesis (33). We demonstrated that ABCC6 knockdown mitigated lipid metabolism and oxygen consumption in HCC cells, which could promote cell proliferation by preventing HCC cells from oxidative damage.

The current study still has several limitations. First, it does not address why *ABCC6* is downregulated in tumor tissues. Moreover, although we demonstrated that ABCC6 regulates the activity of peroxisomes in HCC, further studies are required to determine the underlying mechanism by which ABCC6 suppresses the expression of PPARα and ACOX1. Additionally, our study does not investigate whether ABCC6 is responsible for the resistance to clinically approved HCC drugs, such as sorafenib, regorafenib, and lenvatinib.

In conclusion, ABCC6 depletion inhibits peroxisome activity, protects cancer cells from oxidative damage, and therefore promotes cell proliferation. Our study provides profound insights into the behavior of ABC transporter family genes in various HCC cohorts, identifies ABCC6 as a potential biomarker for early-stage HCC diagnosis, and highlights the significance of ABCC6 in liver cancer progression.

## Data Availability Statement

The datasets presented in this study can be found in online repositories. The names of the repository/repositories and accession number(s) can be found in the article/**Supplementary Material**.

## Ethics Statement

The studies involving human participants were reviewed and approved by the Renji Hospital Ethics Committee. The patients/participants provided their written informed consent to participate in this study. The animal study was reviewed and approved by the Institutional Animal Care and Use Committee of Renji Hospital, Shanghai Jiao Tong University School of Medicine.

## Author Contributions

ZCZ designed the study, performed cellular and molecular experiments, interpreted the data and wrote the manuscript. ZJZ performed the bioinformatics analysis. JW, HZ, and ZX performed cellular and molecular experiments or data analyses. QX conceived the project, supervised the study and revised the paper. All authors discussed the results and commented on the manuscript. All authors contributed to the article and approved the submitted version.

## Funding

This study was supported by National Natural Science Foundation of China (81972205); Major Program of the National Natural Science Foundation of China (92059205); Shanghai Municipal Hospital Three-year-project for Clinical Skills’ Promotion and Innovation (SHDC2020CR5012, SHDC2020CR2003A).

## Conflict of Interest

The authors declare that the research was conducted in the absence of any commercial or financial relationships that could be construed as a potential conflict of interest.

## Publisher’s Note

All claims expressed in this article are solely those of the authors and do not necessarily represent those of their affiliated organizations, or those of the publisher, the editors and the reviewers. Any product that may be evaluated in this article, or claim that may be made by its manufacturer, is not guaranteed or endorsed by the publisher.

## Supplementary Material

Supplementary Table 1 Transcriptome analysis of *ABCC6* knockdown MHCC97H cells versus control cells.
